# Study of the Printing Characteristics of a 3D Printing Solution for the Purpose of Process Optimization

**DOI:** 10.3390/ma18173989

**Published:** 2025-08-26

**Authors:** Shuai Yang, Fei Li, Ya Lu, Xiaoming Xu, Huajie Zhou, Lian Zhou, Yongkang Wei

**Affiliations:** 1 Engineering Technology Innovation Center of Construction and Demolition Waste Recycling, Ministry of Housing and Urban-Rural Development, Beijing University of Civil Engineering and Architecture, Beijing 100044, China; 15210543728@163.com (S.Y.); luyayu@126.com (Y.L.); zla@bucea.edu.cn (L.Z.); m15022579971@163.com (Y.W.); 2 China MCC22 Group Corporation Ltd., Beijing 100062, China; bjcjjsb@163.com (X.X.); zhouhuajie720909@163.com (H.Z.)

**Keywords:** 3D printing, workability, extrudability, supportability, extrusion uniformity, cumulative deformation rate, rheological properties, printing process

## Abstract

Current research and technical standards primarily rely on observational methods to evaluate the printability of 3D printing materials. There is a lack of quantitative assessment metrics for extrudability and supportability, and experimental data cannot be used to characterize extrudability and buildability. Further research is needed. Based on traditional workability parameters (such as flowability), this study explored the influence of printability characteristics and adopted two quantitative indicators—extrusion uniformity and cumulative deformation rate—to comprehensively evaluate material performance from two aspects, while observing the trend of changes in traditional workability indicators and print quality under experimental conditions. The experimental results showed that the extrusion uniformity of 3D-printed mortar initially improved and then gradually deteriorated as flowability increased, and was inversely proportional to dynamic yield stress. The cumulative deformation rate decreases with the improvement of height retention capability and the increase in static yield stress. Through parameter analysis, the optimal printing performance conditions were determined: when the extrusion uniformity is below 3.3% and the cumulative deformation rate is ≤6%, the corresponding dynamic yield stress range is 200 Pa to 800 Pa, and the static yield stress range is 1800 Pa to 3300 Pa. Under these parameters, the mortar exhibits excellent printing performance, including high-layer stacking capability (≥30 layers) and enhanced structural stability. This experiment demonstrates that using these two quantitative indicators can simply and efficiently evaluate the performance metrics of 3D-printed materials, while also revealing the relationship between the workability and printing quality of 3D-printed recycled micro-powder geopolymer materials.

## 1. Introduction

Due to the additive manufacturing of 3D printing, it has significant advantages over traditional manufacturing methods such as not requiring formwork for support, ease of construction, high design flexibility, reduced labor and waste production, and an improved construction environment [1,2,3]. These advantages can lower the negative impact of the construction industry on the environment, reduce carbon dioxide emissions, and have enormous potential for development and application [1,2,3,4]. At the same time, 3D printing technology has higher requirements for building materials. In the concrete 3D printing process, material clogging, interruption, deformation, tearing, and even collapse are common issues. The material must be printable, which includes extrudability and buildability. Extrudability refers to the ability to extrude the material completely and evenly, while buildability refers to the ability of the material to maintain a stable printed shape [5]. Therefore, printing materials need to have excellent printability to achieve continuous printing, no cracking, and no collapse [6,7,8,9]. In other words, to ensure the success of 3D printing construction, it is necessary to clearly define the material ratio while researching the characterization standards used to demonstrate printability [10,11].

Numerous studies have been conducted by scholars worldwide on the printability of 3D printed concrete. Although it is currently common practice to characterize the printability of 3D-printed concrete using liquidity, rheological properties, and thixotropy, there are slight differences in the specific test methods and characterization parameters used by different scholars [12]. Some scholars judge whether printability is achieved by comprehensively evaluating the properties of the mortar. Xu et al. [13] noted in their experiments that the printability of mortar can be characterized by consistency and flowability. To better characterize printability using consistency and flowability, a linear fit was performed on the data, yielding the fitting equation y = 1.2011x + 104.5023, R^2^ = 0.9359. It was determined that when the viscosity range is 49.0–85.2 mm and the flowability is 162.3–205 mm, the slurry exhibits good printability. Liu et al. [14] printed single-layer strips, cut the hardened strips every 100 mm after 24 h of curing, and selected three different positions of the printed cross-sections for analysis. They evaluated the uniformity and flatness of the printed strips by calculating the median of the slope of the fitted line and the median of the sum of squares of the residuals of the fitted line. When both values were small, it indicated good printability. Most researchers evaluate extrudability using flowability, which is considered a simple way to assess the extrudability of the investigated mortar. If the extruded strip meets the requirements of continuity and consistency, it indicates that the slurry meets the requirements of extrudability [15]. Currently, a large number of studies have shown that when fluidity is basically maintained between 160 and 210 mm, it has a good extrusion effect [5]. Some scholars also began to focus on the performance of the mortar after printing. Le et al. [16] set the standard for extrudability as the ability to extrude continuous strips with a total length of 4500 mm without clogging or breaking by using a nozzle width of 9 mm. Ali et al. [17] conducted numerous experiments to print strips with a width of 38.1 mm and concluded that an error of less than 10% in the target width is acceptable, indicating good extrudability. Nair et al. [18] defined the flow rate ratio using the print width and print height, i.e., the ratio of the nominal cross-sectional area of the print to the design cross-sectional area, to determine the extrusion quality of the slurry print. A large deviation from 1.0 in the flow rate ratio indicates poor extrudability, while a value close to 1.0 indicates good extrudability. Biranchi et al. [19] determined the extrudability of 3D printing materials based on rheological parameters, which depended largely on the chemical composition of the material and the equipment used to measure the properties. There are also relevant evaluation methods for buildability, mainly direct methods and indirect methods [20]. Ashrafi et al. [21] evaluated the supportability of 3D printing materials by testing the maximum print height. Panda et al. [22] assessed the supportability of the materials by the highest number of print layers, and verified that high static yield stress effectively improved the supportability of printing materials. Nerella et al. [23] proposed a practical criterion for assessing buildability by printing a scaled-down model of the desired structure and determining the time interval between layers based on the economic feasibility of the target structure. In addition, some scholars have indirectly determined buildability by using unconfined uniaxial compression tests (UUCT), which can measure the compressive strength and Young’s modulus of 3D-printed slurry at different ages, as well as calculate the rate of change in these parameters over time [24]. However, although the direct testing method is simple, it requires a large amount of work, while the indirect testing method has the disadvantage of large data dispersion [15,20]. Therefore, it is still necessary to establish a set of standardized parameters to assess buildability. In summary, extrudability and supportability are important performance indicators for evaluating the printability of 3D printing pastes. There is still a lack of simple and accurate quantitative evaluation methods for the printability of 3D printing slurries, including extrudability and supportability [25,26,27]. This paper takes 3D-printed recycled micro-powder geopolymer mortar as an example to quantitatively evaluate extrusion and buildability properties through extrusion uniformity and a cumulative deformation rate, respectively. It analyzes the changes in the corresponding relationships and ultimately proposes a parameter range suitable for 3D-printed recycled micro-powder geopolymer mortar, providing a possible new method for 3D-printable testing.

## 2. Raw Materials and Experimental Methods

### 2.1. Raw Materials

**Figure 1:**
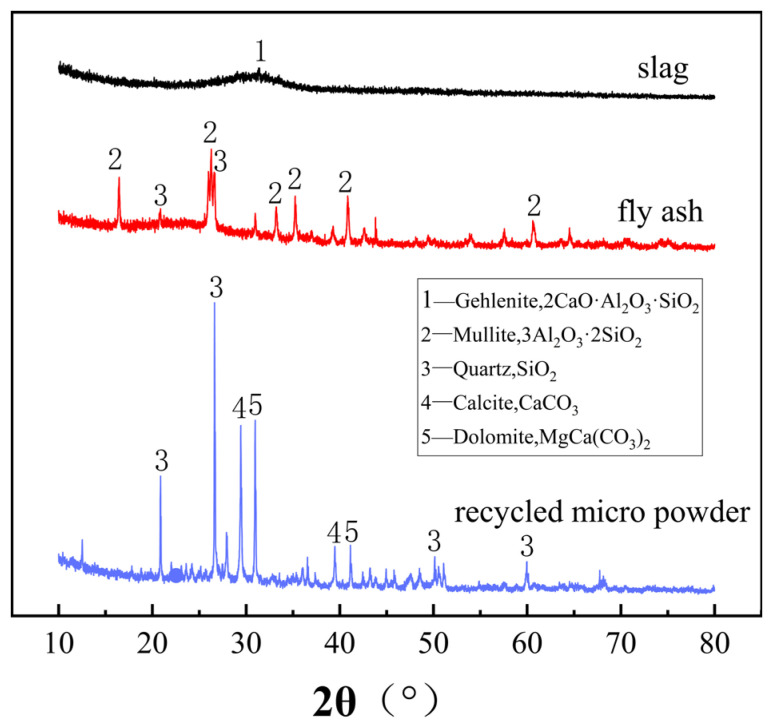
XRD spectra of mineral powder, fly ash, and construction waste micro-powder.

The recycled micro-powder used in this paper was collected from the bag filter of the construction waste crushing production line. The mineral powder and fly ash were provided by JinTai Cheng Environmental Protection Technology Co., Ltd. in Xingtai, Hebei Province, China. Among them, particles with a particle size ≤ 21.8 μm accounted for 50.0% of the overall recycled micro-powder, and particles below 10 μm accounted for 30.3%. The XRF analysis results of the three powder raw materials are shown in Table 1, and the XRD analysis results are shown in Figure 1.

**Figure 2:**
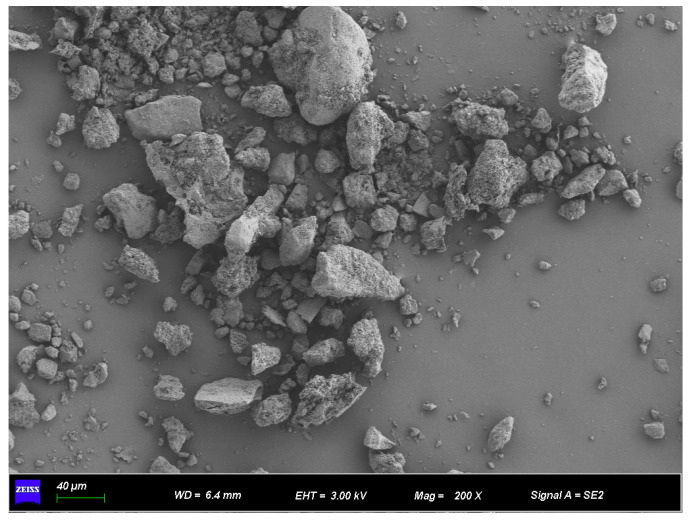
Microscopic morphology of construction waste micro-powder.

The SEM analysis of recycled micro powder, as shown in Figure 2, reveals that the particle morphology is mostly irregular and lumpy with sharp edges. Some particles have rough surfaces and contain numerous pores and cracks, and their cross-sections are uneven [28].

The aggregates used in this paper are washed sand from Beijing Yugou Group Co., Ltd. in Beijing, China, with particle sizes of 20–40 mesh and 40–70 mesh in a ratio of 3:2.8. The alkali activator is a composite of liquid sodium silicate (Na_2_O·nSiO_2_) and analytical grade NaOH with a purity of 96%, produced by Jiashan Yourui Refractory Materials Co., Ltd. in Zhejiang Province, China, with a modulus fixed at 1.4. The solid content of sodium silicate was 43.74%, with SiO_2_ content of 29.99% and Na_2_O content of 13.75%, at a concentration of 50°Bé. The admixtures used in this paper include polyvinyl alcohol (PVA), hydroxypropyl methylcellulose ether (HPMC), hydroxypropyl cellulose (HPC), redispersible latex powder, polypropylene fibers (PP fibers), ZnCl_2_ (99% purity, analytical grade), nano-SiO_2_, and a water-retaining and thickening agent with a HPMC/HPC/latex powder ratio of 4:1:10.

### 2.2. Experimental Methods

#### 2.2.1. Workability Test Method

The flowability of mortar was determined according to the test method of cement mortar flowability in GB/T 2419-2005 [29]. The flowability of the diameter in two mutually perpendicular directions was measured using a jump table test, and the average value was calculated. The entire process was completed within 6 min.

#### 2.2.2. Rheological Test Method

The experiment was conducted using a BROOKFIELD RST touch screen rheometer (Middleboro, MA, USA) with a maximum torque of 100 mNm and a speed range of 0.01–1300 min^−1^. The spindles use a coaxial cylinder spindle geometry with specifications of CCT-40, a viscosity range of 0.0003–27.6 Pa·s, and a shear rate of 0.0215–2.79 k s^−1^.

The rheological curve was determined as follows:

Dynamic yield stress: (1) the slurry was pre-sheared for 1 min at a shear rate of 30 s^−1^; (2) rested for 1 min; (3) the shear rate increased from 0 s^−1^ to 10 s^−1^ in the next 200 s by gradient shear rate; and (4) the shear rate decreased from 10 s^−1^ to 0 s^−1^ in the next 200 s by gradient shear rate.

Static yield stress [30]: (1) the slurry was pre-sheared for 30 s; (2) rested for 10 min; and (3) the shear rate was set at 0.1 s^−1^ for a uniform test of 180 s.

The dynamic and static yield stresses were described by the mathematical expression of the modified Bingham (MB) model [31,32,33].

#### 2.2.3. Printability Test Method

**Figure 3:**
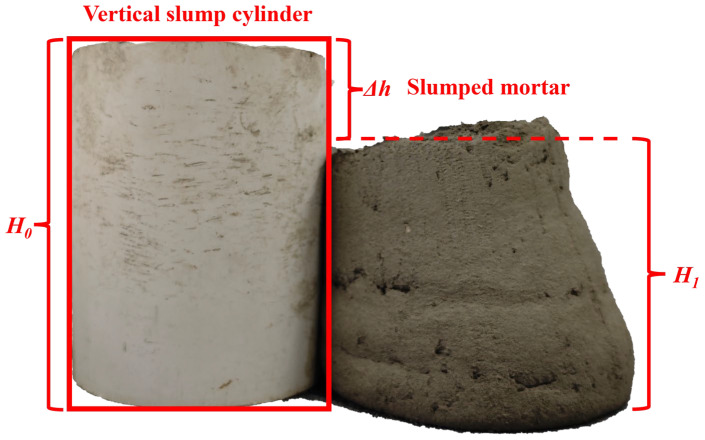
Schematic diagram of the method for calculating high retention rate.

The height retention rate refers to the ratio of the difference between the height of the retained paste and that of the vertical slump cylinder to the height of the vertical slump cylinder itself [7,34]. It is used to investigate the deformation caused by the material’s own weight during the printing process, which can quickly and easily evaluate the quality of the material ratio and its support. The schematic diagram is shown in Figure 3, and the calculation method is presented as Equation (1). The prepared paste was poured into the cylinder in three portions, and a pestle was used to tamp it in 25 times until the cylinder was filled. Then, the vertical drop height cylinder was slowly lifted, and the height of the collapsed paste was measured after waiting for the paste to freely collapse until it no longer deformed. As shown in Equation (1), the difference between the height of the vertical drop height cylinder and this height (*H*_1_) divided by the height of the vertical drop height cylinder *(H*_0_) is the height retention rate of the paste.

(1) $$h=\frac{H_{1}}{H_{0}}$$

### 2.3. Three-Dimensional Printer Parameter Settings

A concrete (mortar) 3D printing system (desktop level) from Hangzhou Jianyan Huace in Zhejiang Province, China was used for the 3D printer in this study. All experiments requiring the use of a 3D printer employed fixed equipment parameters. Through preliminary experiments combining extrusion speed and printing feed speed, the optimal printing results were achieved with a fixed nozzle diameter of 20 mm, a horizontal printing head movement speed of 50 mm/s, a vertical printing head lifting speed of 5 mm/s, an extruder speed of 1.5 r/s, and a slicing mode of layer-by-layer slicing.

### 2.4. Experimental Method and Mixture Proportions

#### 2.4.1. Experimental Mixture Proportions

This experiment used a fixed ratio of furnace slag to fly ash by mass, with a ratio of 7:3. The binders include slag, fly ash, and recycled micro powder. The mass ratio of cementitious materials to sand was 0.8, the polypropylene fiber content was 0.7%, the recycled microfine content was 10%, the polyvinyl alcohol (PVA) content was 0.2%, and the zinc chloride (ZnCl_2_) content was 1.5%. The content of nano-silica is 1%, and the content of the water-retaining thickening agent is 0.7%. Experiments were conducted to test the performance of 3D-printed mortar by adjusting the mass ratio of water to binder from 0.30 to 0.50, increasing by 0.05 each time. The experimental mixtures are shown in Table 2.

#### 2.4.2. Evaluation Method of Extrudability

Extrudability refers to the ability of 3D printed mortar extruded by the printing head, meeting the performance requirements of continuous printing construction. The ease of extrusion is used to evaluate the extrudability of the investigated mortar. Currently, there is no quantifiable evaluation index for the extrudability of 3D printed mortar. This experiment attempts to establish a quantitative method for evaluating the extrudability of mortar. Based on the existing standard T/CECS 786-2020 [35] “Technical Specifications for Concrete 3D Printing Technology,” by observing whether the printed strips are continuous and uniform, free of blockages, and without obvious cracks, the uniformity index of extrusion is proposed to ensure continuous and unblocked extrusion. The specific experimental method is as follows:

Continuity of Extrusion: During the printing process, it is stipulated to observe whether there is obvious tear, block, or self-flow without the need for extrusion pressure in the continuous printing of a 4800 mm strip. If so, it is considered that the mortar has poor extrusion continuity and can be directly evaluated as poor extrudability. If not, the extrusion continuity is satisfied, and the extrusion uniformity is evaluated.

**Figure 4:**
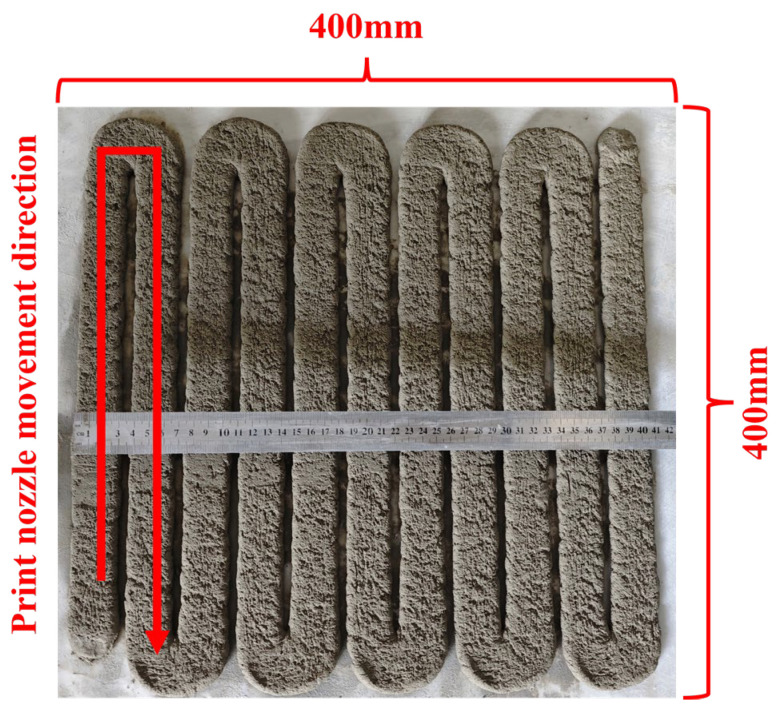
Schematic diagram of extrudability testing for print paths.

Uniformity of Extrusion: After the mortar is actually printed on the machine according to Figure 4, the width of each vertically printed strip is measured at three different locations, and the coefficient of variation in the width is calculated. The average value of the coefficient of variation in the three strips is used to characterize the extrusion uniformity of the material. The smaller the coefficient of variation, the better the extrusion uniformity and the extrudability, and vice versa. The calculation formulas are shown in Equations (2) and (3).

$$C_{k}=\frac{S}{\bar{x}}\times 100\%=n\sqrt{\frac{\sum_{i=1}^{n}{\left(x_{i}-\bar{x}\right)}^{2}}{n}}/{(x}_{1}+x_{2}+\cdot \cdot \cdot \cdot \cdot \cdot \cdot +x_{n})\times 100\%$$

(2) (K=1,2,3;  n=11)

(3) $$\bar{C}=(\sum_{k=1}^{3}C_{k})/3\;\left(K=1,2,3\right)$$

where *C_k_* is the coefficient of variation in the width of the vertical printed stripes; *S* is the standard deviation of the width of the vertical printed stripes (mm); $\bar{x}$ is the average width of the vertical printed stripes (mm); *x_i_* is the width of a specific vertical printed stripe (mm); *n* is the number of vertical printed stripes; and $\bar{C}\;$ is the average coefficient of variation in the width of the vertical printed stripes.

#### 2.4.3. Supportability Evaluation Method

Supportability refers to the ability of the 3D printed mortar to maintain shape stability after printing. The main sources of deformation of the printed mortar are the self-gravity of the printed mortar, the pressure of the upper layer of the printed mortar on the lower layer, and the extrusion pressure and downward pressure of the printer nozzle on the surface of the mortar. However, T/CECS 786-2020 [35] “Technical Specifications for 3D Printing of Concrete” only stipulates that the shape of the extruded strips should remain stable and not collapse, but lacks quantitative analysis of the testing methods for support. Therefore, this experiment attempts to establish a quantitative evaluation method for supportability based on this basis.

**Figure 5:**
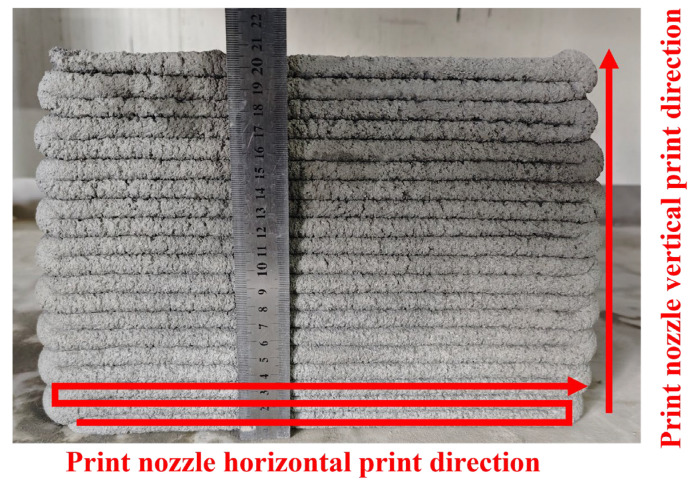
Schematic diagram of supportability testing for print paths.

Print height cumulative deformation rate: The 3D printing slurry is prepared and stacked according to the printing path shown in Figure 5. When the number of printed layers is ≥20, the maximum actual height of the printed wall is measured by a ruler, and the cumulative deformation rate is calculated according to Equation (4). The smaller the cumulative deformation rate, the stronger the slurry’s ability to resist deformation and the better its support. In addition, when the number of printed layers is <20, it is considered that the slurry has no support, and the cumulative deformation rate is not calculated.

(4) $$\Delta h=\frac{H-H^{\prime }}{H}\times 100\%$$

where Δ*h* is the cumulative deformation rate of the printed wall height (%); *H* is the theoretical printed wall height (mm); and *H′* is the actual printed wall height (mm).

## 3. Experimental Results and Analysis

### 3.1. Investigation on Extrudability of 3D Printed Mortar

#### 3.1.1. Effect of Flowability on Extrudability

To achieve good printability, the slurry must have the ability to be extruded smoothly through the screw and maintain a good slurry state after extrusion. Excellent extrudability is the foundation for printing higher and more stable structures, and it is also a prerequisite for the slurry to have supportability.

**Figure 6:**
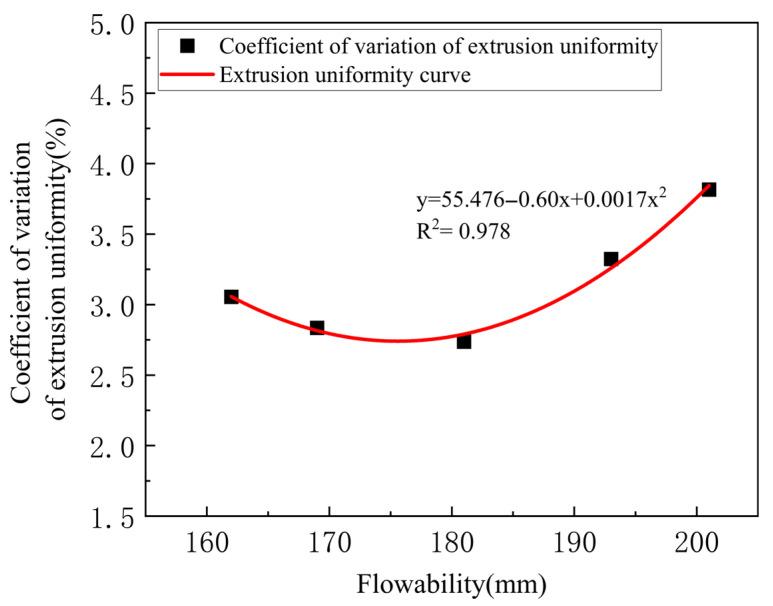
Curve fitting of the relationship between flowability and extrusion uniformity of mortar.

**Figure 7:**
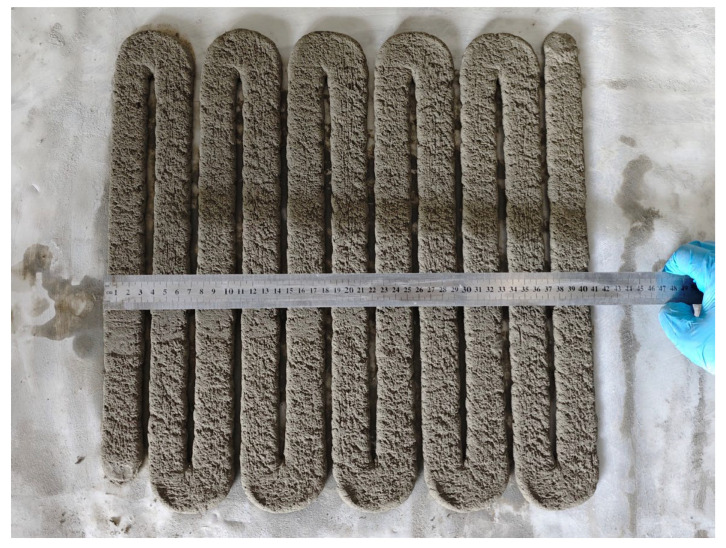
Mortar with excellent extrusion uniformity.

To investigate the relationship between flowability and extrudability, extrudability tests were conducted on the samples prepared in Table 2. The flowability and extrusion uniformity of the experimental results were subjected to second-order linear fitting. As shown in Figure 6, the fitting coefficient is greater than 0.95, indicating that the experimental data is well aligned with the fitting trend. This allows for an accurate determination of the trend of the effect of fluidity on extrudability. When the fluidity of the slurry is below 162 mm or above 201 mm, continuous printing and extrusion continuity cannot be achieved, indicating a lack of extrudability. When the flowability of the slurry is between 162 mm and 201 mm, the coefficient of variation in the extrusion uniformity of the 3D printed slurry decreases and then increases with the increase in flowability. This is because when the flowability is less than 162 mm, the slurry is too dry and hard to fully and uniformly wrap around the aggregate, increasing the internal friction and making the paste difficult to extrude. When the flowability is greater than 193 mm, the extrusion screw and nozzle produce a squeezing effect on the paste, causing “splashing” due to the thin consistency of the paste under pressure, making it difficult to extrude uniformly [36]. As shown in Figure 7, when the flowability is between 162 mm and 193 mm, the coefficient of variation in the extrusion uniformity is small, indicating that the change in the width of the printed stripes in this flowability range is small, and the extrusion is more uniform.

#### 3.1.2. Effect of Dynamic Yield Stress on Extrudability

**Figure 8:**
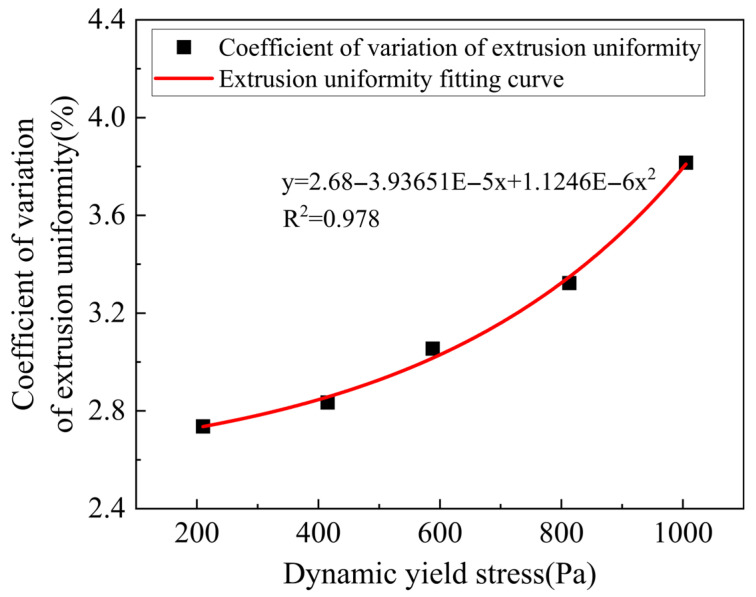
Curve fitting of the relationship between dynamic yield stress and extrusion uniformity of mortar.

Dynamic yield stress is closely related to the flow state of mortar before and after extrusion, which is a key influencing factor in ensuring workability and extrudability. It characterizes the minimum shear stress that the material needs to overcome to maintain its flow state. The smaller the dynamic yield stress, the weaker the ability of the slurry to resist the shearing force of the extrusion screw, and the easier it is to extrude [37,38,39,40]. To investigate the relationship between the dynamic yield stress of mortar and its extrudability, a second-order linear fitting was performed on the dynamic yield stress and the coefficient of variation in extrusion uniformity in the rheological test results of the mortar in Table 2. The fitted curve is shown in Figure 8.

As depicted in Figure 8, when the dynamic yield stress of the mortar is less than 200 Pa or greater than 1000 Pa, it cannot be continuously printed and does not meet the requirements for extrusion continuity. However, when the dynamic yield stress of the mortar is between 200 Pa and 1000 Pa, it can be smoothly extruded, satisfying the requirement for extrusion continuity. However, the fitted curve shows that the coefficient of variation in mortar extrusion uniformity increases with increasing dynamic yield stress. When the dynamic yield stress is less than or equal to 800 Pa, the coefficient of variation in the extrusion uniformity of the printed strip is small, with a maximum value of only 3.3%, and the width of the printed strip is relatively uniform. When the dynamic yield stress is greater than 800 Pa, the coefficient of variation in the extrusion uniformity of the printed strip increases significantly, with a maximum variation coefficient of up to 4.8%, and the extrusion is less uniform. Therefore, it is believed that the range of dynamic yield stress between 200 Pa and 800 Pa is favorable for the extrudability of mortar.

### 3.2. Three-Dimensional Printing Mortar Supportability Investigation

#### 3.2.1. Relationship Between Height Retention and Supportability

Another important indicator affecting the printability of the slurry is its supportability. Since the 3D printing process has no template support during printing and relies only on the self-bearing capacity of the material and the bite force between each layer of the slurry to achieve upward stacking printing, the supportability of the slurry is particularly important [41,42,43]. The larger the height retention rate, the smaller the deformation caused by the self-gravity of the slurry, which is more conducive to the stability of the printed structure.

**Figure 9:**
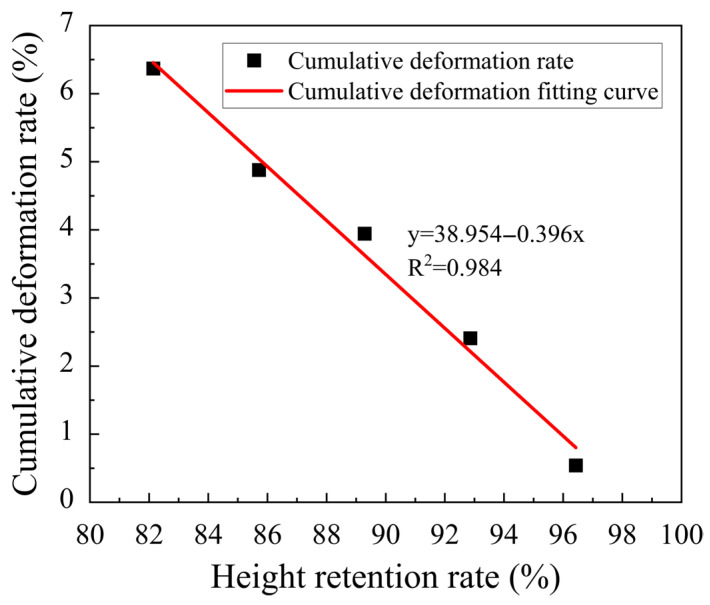
Curve fitting of the relationship between the high retention rate and cumulative deformation rate of the mortar.

As mentioned above, a slurry with poor extrudability often breaks during the printing process, which cannot provide a stable, flat, and uniform lower layer slurry for the upper layer slurry, resulting in partial collapse of the upper layer slurry due to the rupture of the lower layer slurry. This causes the inconsistence of the distance between the slurry and the nozzle, and the upper layer slurry cannot be evenly squeezed onto the lower one, resulting in uneven stress on the lower layer slurry and the instability and collapse of the printed structure, which is not conducive to supportability. Therefore, based on the extrudability test, the relationship between height retention and supportability was further explored by conducting supportability testing experiments on the proportions in Table 2, and the height retention rate and cumulative deformation rate in the experimental results were subjected to second-order linear fitting as shown in Figure 9.

**Figure 10:**
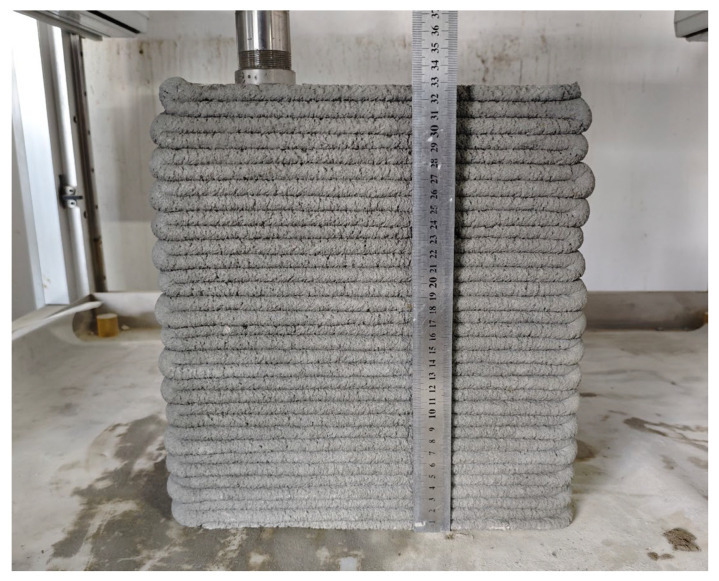
Mortar with excellent supportability.

From Figure 9, it can be seen that there is no support when the height retention rate of the slurry is less than 82.1% or greater than 96.4%. This is attributed to the fact that when the height retention rate is too low, the slurry undergoes excessive deformation, making it difficult to continuously print more than 20 layers. Conversely, when the height retention rate is too high, the slurry is difficult to extrude and cannot meet the extrudability requirements. When the height retention rate is between 82.1% and 96.4%, there is a negative correlation between the cumulative deformation rate of the 3D printed wall and the height retention rate of the slurry, that is, the greater the height retention rate, the stronger the slurry’s ability to resist external pressure and maintain its shape. When the height retention rate is greater than or equal to 89.3%, the slurry can be continuously printed for more than 30 layers with a cumulative deformation rate of less than 4%, and the support is good, as illustrated in Figure 10. However, when the height retention rate is less than or equal to 85.7%, the cumulative deformation rate exceeds 6%, and the slurry’s ability to resist the gravity and nozzle extrusion force is weak during the printing process, making it susceptible to deformation and instability, resulting in poor support. Therefore, when the height retention rate of the slurry is between 96.4% and 85.7%, it exhibits good supportability.

#### 3.2.2. Relationship Between Static Yield Stress and Supportability

Static yield stress represents the maximum shear stress that a material must overcome to undergo plastic flow. It may have a good correlation with the shape stability of 3D printed slurry during the static settling stage after extrusion. A suitable range of static yield stress not only has a minimal negative impact on the extrudability of mortar, but also significantly enhances its supportability and resistance to external pressure, thus reducing deformation and preventing the instability and failure of 3D printed structures [44,45,46].

**Figure 11:**
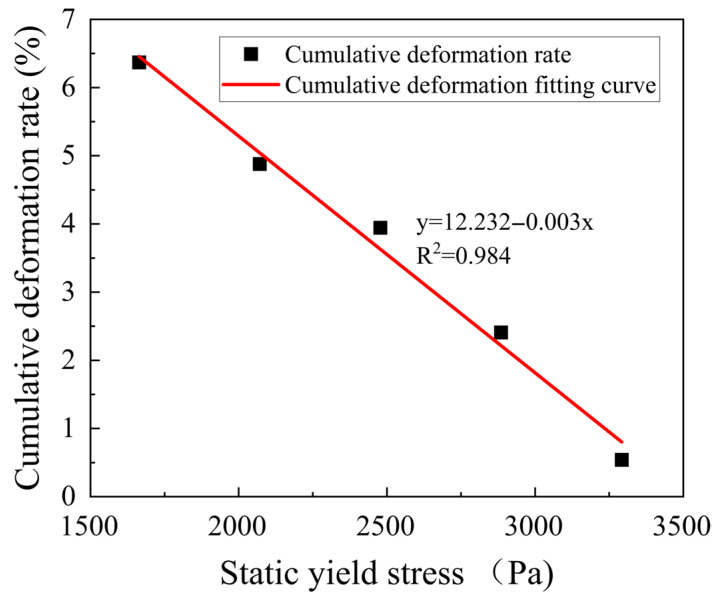
Curve fitting of the relationship between static yield stress and the cumulative deformation rate of mortar.

To investigate the relationship between the static yield stress and supportability of the mortar, the static yield stress values obtained from the test results of Table 2 were fitted with the cumulative deformation rates through a quadratic linear regression. The resulting fitted curves are shown in Figure 11. When the static yield stress is less than 1600 Pa or greater than 3300 Pa, the slurry is prone to flow and undergoes large deformation, making it difficult to print more than 20 layers continuously or overcome the large shear force required for slurry flow, which results in poor extrudability and lack of supportability. The data for the interval where the static yield stress is greater than 1600 Pa and less than 3300 Pa is summarized in Figure 11. On the other hand, it can be seen that the cumulative deformation rate of the 3D printed wall decreases significantly with an increase in both variables, indicating that an increase in the static yield stress can effectively control the deformation of the printed wall, enhancing the ability of the slurry to resist deformation, and improving its supportability. Furthermore, when the static yield stress of the slurry is less than 2484 Pa, the cumulative deformation rate of the printed wall decreases rapidly, but slows down as the static yield stress increases. According to some studies [47], this may be attributed to the limit bearing capacity of the bottom layer of the slurry, which has a maximum deformation value at a certain limit when subjected to pressure from the upper layers. The closer the deformation value is to the limit, the smaller the deformation of the slurry. However, when the upper pressure exceeds the limit bearing capacity of the slurry, the deformation change in the slurry will experience a sudden mutation, causing instability and failure. Therefore, based on the analysis, when the static yield stress is within the range of 1800 Pa to 3300 Pa, the cumulative deformation rate of the 3D printed regenerated micronized geopolymer mortar wall is less than or equal to 6%, and when the printing layer is higher (≥30 layers), it exhibits good supportability.

## 4. Conclusions

This study investigates the correlation between traditional rheological properties (including flow characteristics) and printability parameters of 3D printing materials. Through comprehensive analysis, we adopted an evaluation method that combines extrusion and supportability indicators to assess material printability. In this experimental context, by observing the fitting trends between material performance indicators and printing performance indicators, we summarized patterns and arrived at three main findings:

(1) The method proposed in this study, which uses the extrusion uniformity and cumulative deformation rate of regenerated micro-powder geopolymer mortar to assess the extrusion and support properties of 3D-printed materials, has significant advantages over existing printability assessment methods and can be used to evaluate the extrusion and buildability of 3D-printed mortars. Under the experimental conditions of this study, when the recycled micro powder content is 10% and the nano-silica content is 1%, the extrusion and buildability of the mortar can be effectively improved.
(2) The extrusion uniformity of 3D printing materials exhibits a non-monotonic relationship with mortar flowability. As flowability increases, uniformity initially decreases before increasing, and improves with the increase in dynamic yield stress. Under the experimental parameters in this study, when the dynamic yield stress ranges from 200 Pa to 800 Pa, extrusion uniformity can be maintained below 3.3%.
(3) The cumulative deformation rate is inversely proportional to the height retention rate and static yield stress, decreasing as the height retention rate and static yield stress increase. The experimental results indicate that controlling the static yield stress within the range of 1800 Pa to 3300 Pa can effectively keep the cumulative deformation rate at ≤6%. At this point, with a high number of printed layers (≥30 layers), the mortar exhibits good buildability.

Under the experimental conditions of this study, the research proposed specific ranges for extrusion uniformity and cumulative deformation rate control within the specified dynamic yield stress and static yield stress ranges. The results emphasize that mathematical fitting regression analysis methods provide a new approach for assessing the printability of 3D-printed mortar. However, this study has limitations due to the relatively fixed experimental parameters, and the fitted data only represents trends with limited data volume. Future research should focus on printer parameters and other materials, by altering the nozzle diameter, printing parameters, and printing materials, to enrich experimental data and expand the applicability of extrusion uniformity and cumulative deformation rate assessments for evaluating the printability of a 3D-printed mortar.

## Figures and Tables

**Table 1:** The chemical composition of cementitious materials/%.

Inspection Materials	CaO	SiO_2_	Al_2_O_3_	Fe_2_O_3_	MgO	SO_3_	Na_2_O	K_2_O	Others
mineral powder	47.1	25.9	12.7	0.3	6.7	2.8	0.4	0.4	3.7
fly ash	3.8	52.6	33.3	5.1	0.6	0.5	0.3	1.3	2.5
recycled micro-powder	12.4	54.7	15.9	6.5	3.3	0.7	2.0	2.9	1.6

**Table 2:** Admixture proportion for the printability performance testing of 3D printing mortar.

Sample	Materials/g						Admixtures/g				Alkali Activator/g	
	Slag	Fly Ash	Water	Sand	PP Fiber	Recycled Micro Powder	PVA	Water Retaining Thickener	ZnCl_2_	Nano-Silica	Na_2_O·nSiO_2_	NaOH
S-0.30	283.5	121.5	135	562.5	3.15	45	0.9	3.15	6.75	4.5	164.61	8.08
S-0.35			157.5									
S-0.40			180									
S-0.45			202.5									
S-0.50			225									

## Data Availability

The original contributions presented in this study are included in the article. Further inquiries can be directed to the corresponding author.
